# TrOn: An Anatomical Ontology for the Beetle *Tribolium castaneum*

**DOI:** 10.1371/journal.pone.0070695

**Published:** 2013-07-30

**Authors:** Jürgen Dönitz, Daniela Grossmann, Inga Schild, Christian Schmitt-Engel, Sven Bradler, Nikola-Michael Prpic, Gregor Bucher

**Affiliations:** 1 Johann-Friedrich-Blumenbach Institute of Zoology and Anthropology, Göttingen, Germany; 2 Department of Bioinformatics, University Medical Center Göttingen, Göttingen, Germany; Oxford Brookes University, United Kingdom

## Abstract

In a morphological ontology the expert’s knowledge is represented in terms, which describe morphological structures and how these structures relate to each other. With the assistance of ontologies this expert knowledge is made processable by machines, through a formal and standardized representation of terms and their relations to each other. The red flour beetle *Tribolium castaneum*, a representative of the most species rich animal taxon on earth (the Coleoptera), is an emerging model organism for development, evolution, physiology, and pest control. In order to foster *Tribolium* research, we have initiated the *Tribolium* Ontology (TrOn), which describes the morphology of the red flour beetle. The content of this ontology comprises so far most external morphological structures as well as some internal ones. All modeled structures are consistently annotated for the developmental stages larva, pupa and adult. In TrOn all terms are grouped into three categories: Generic terms represent morphological structures, which are independent of a developmental stage. In contrast, downstream of such terms are concrete terms which stand for a dissectible structure of a beetle at a specific life stage. Finally, there are mixed terms describing structures that are only found at one developmental stage. These terms combine the characteristics of generic and concrete terms with features of both. These annotation principles take into account the changing morphology of the beetle during development and provide generic terms to be used in applications or for cross linking with other ontologies and data resources. We use the ontology for implementing an intuitive search function at the electronic iBeetle-Base, which stores morphological defects found in a genome wide RNA interference (RNAi) screen. The ontology is available for download at http://ibeetle-base.uni-goettingen.de.

## Introduction

Gene knock-down by RNA interference (RNAi) [1] has made it possible to study gene function in many different arthropod model systems. Large scale screens are under way and are used to determine the function of thousands of genes. Due to the ease of culture and its amenability to forward and reverse genetic methods, the red flour beetle *Tribolium castaneum*, a representative of the most species rich taxon on earth, the Coleoptera, has been developed into an insect model system second only to *Drosophila*. It is used for research of different topics such as evolution and development of trunk, head and brain, physiology and pest control [2]–[9]. In the ongoing large scale RNA interference (RNAi) screen “iBeetle”, genes have been systematically knocked down in the red flour beetle *Tribolium castaneum*. Upon injection of dsRNA into larvae and pupae, the respective gene function is knocked down and the resulting morphological phenotypes have been documented in an electronic database using a defined vocabulary. The focus has been on developmental defects during embryogenesis and metamorphosis, on muscle development and on oogenesis. About 5.000 genes have been screened so far (Bucher unpublished). In such projects phenotypic data for thousands of genes is generated which requires a controlled vocabulary describing the wildtype and phenotypic morphology.

In textual form the morphology of *Tribolium* is described in large parts in the text book “The biology of *Tribolium*” from Sokoloff [10]. This work is accepted as reference for the *Tribolium* anatomy. However, being a printed medium, the valuable knowledge cannot be included in automatic data processing or online databases. Some details with respect to morphological descriptions are not covered by Sokoloff but were named when the need arose and the respective information is distributed in various publications. Examples are aspects of central nervous system morphology [8], a set of setae and bristles present on walking legs of first instar larvae [11] and a set of setae and bristles marking the dorsal part of the *Tribolium* head [12], [13].

**Figure 1:**
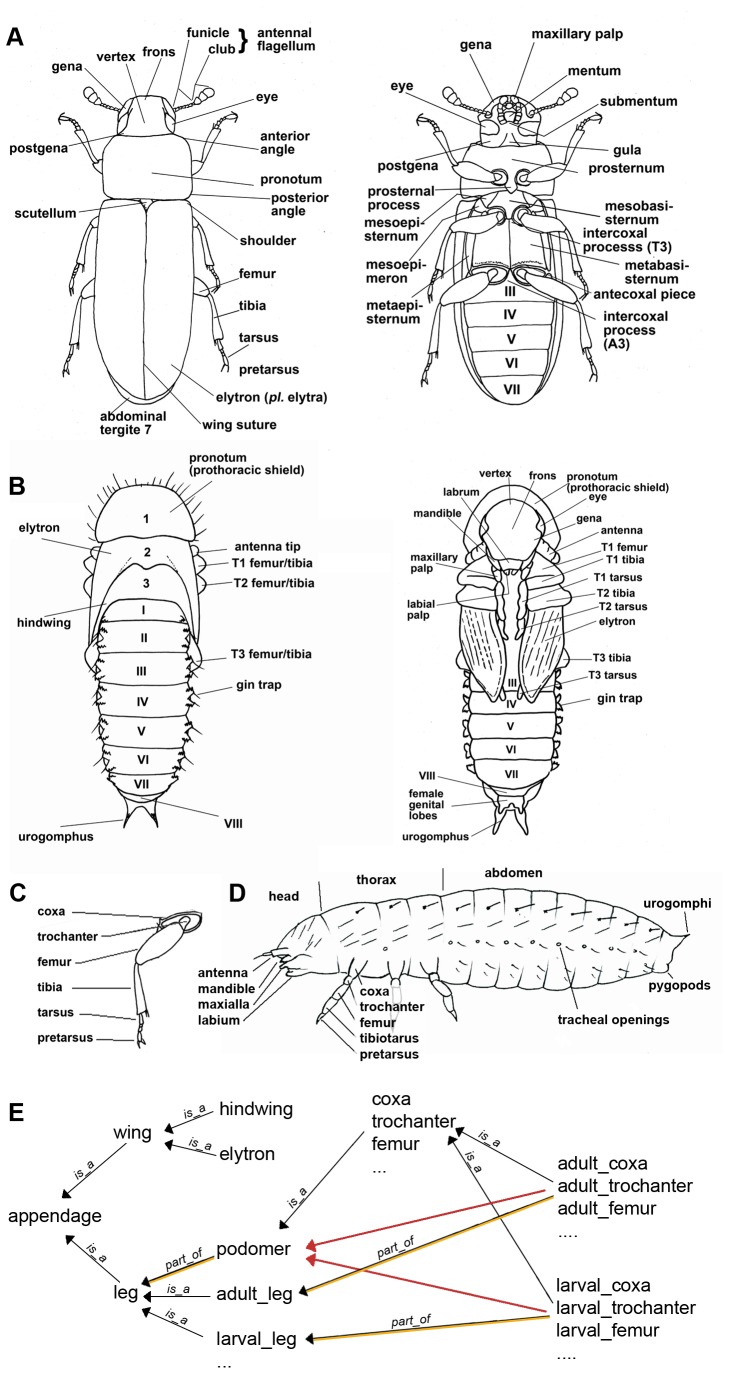
Morphology of the red flour beetle *Tribolium castaneum*. Morphological terms as represented in TrOn are given for three life stages: adult A), pupa B) and first instar larva D). The podomers of the adult walking legs are shown in C). E) Example for the relations is_a and part_of between terms of the ontology. Note that for simplicity not all terms and not all connections are shown. There are several types of *appendages*, for instance *legs* and *wings*. There are two types of *wings* (*elytron* and *hindwing*). The *legs* are present at all life stages (e.g. adult_leg *and* larval_leg). Legs are composed of several *podomeres* (see panel C) and each podomer is part_of a *leg*. Each life stage has legs which contain podomeres. Hence, the adult_trochanter has several relations: it is_a *trochanter* and it *is_a* podomer and it is part_of the adult_leg. In an ontology, is_a relations can be used to infer indirect connections. For instance, the adult_trochanter is_a trochanter. In addition, the trochanter is defined as a podomer. As consequence, also the adult_trochanter is regarded as a podomer although this relation has not been defined directly in the ontology (see red arrow). When implemented in databases, such an ontology can help to make searches more intuitive. A search for “*appendage*” would reveal datasets, with *wing* or *larval_trochanter* phenotypes. A search for datasets affecting *podomer* or *leg* would not consider datasets with annotated wings.

Additional structures have been named in order to allow annotation in the iBeetle screen, like for instance the anterior angle of the pronotum (Klingler, Bucher unpublished). An overview about the morphology of *Tribolium castaneum* at larval, pupal and adult life stages is given in Figure 1 together with labels for important anatomical structures.

Due to the distribution of this information in several publications, *Tribolium* research can at the moment not build on a central, searchable and authoritative repository. As the *Tribolium* model system is gaining popularity it is the right moment to initiate such a resource before different terms and definitions of the same structure get established in parallel like the example of the paramere of the hymenoptera as shown by Yoder at al. [14]. During their efforts to build an ontology which covers the lineage of the Hymenoptera (HAO) they faced the difficulty, that the term paramere was used quite different in the available publications. Not only a smaller or larger set of structures of the male genitalia were labeled as paramere, but also the publications which restrict the term to a small subset of structures did not always agree on the same structures. Moreover, even if the morphological knowledge is mapped in a one-to-one correspondence to terms and their definitions, still the relations between them would not be accessible for automatic processing.

The state of the art method to collect and organize a complex resource like that is the use of ontologies. Ontologies are used to represent an expert’s knowledge in a formal and standardized way. Each item or concept of the real world is represented in the ontology by one *term*. An ontology term identifies unambiguously one item and describes it. Therefore, a term has a name and may have a list of synonyms, a definition or other properties which help to describe the term, like references to other resources. Furthermore an ontology does not only define a collection of terms, but also a set of *relation types*, to describe the connections between the terms. A highly simplified depiction of an ontology of terms related to walking legs is shown in Fig. 1E (see legend for further details).

The most important connection is the is_a relation type. Except for the *root term*, each term has one or more *parents* and may have several *child* terms. Following the concept of *inheritance*, a child term is a specialization of its parents distinguished by a differentiation specific for the child (*genus-differentia* [15]). For example, the antenna is a child of the parent term appendage and is connected to its parent by an is_a relation. Hence, it inherits the characteristics of the parent (i.e. that it is a moveable outgrowth like other appendages) but it is special in that the antenna is located on the dorsal head and has functions different from other appendages. Besides of this term hierarchy, an ontology can define other relation types, most common is part_of. The part_of relation type facilitates the representation of logical interconnection between morphological terms, which are clear to the researchers but are not covered by the term hierarchy. For instance, the antenna can be linked to the head with a part_of relation while it is connected to appendage with the is_a relation. A child term is always a specialization of its parents and inherits their relations. E.g. larval_antenna is a child of larval_appendage and inherits the part_of relation from larval_appendage to larva. While this inheritance of relations is always defined along the axis of the class hierarchy it is not mandatory for the other relation types of an ontology. If such a relation type is not explicitly defined as transitive the term’s relations are not inherited along the non-is_a relations. This definition is made at the level of the relation type and is obligatory for all relations of this kind. To reuse the example of the antenna, the flagellum has a part_of relation to the term antenna and a is_a connection to its parent term multicellular_tissue. If the relation type in this case would be defined to be transitive, flagellum would be part of the head due to two part_of relations: The one between flagellum and antenna and between antenna and head. Otherwise the assertion that the flagellum is a part of the head cannot be deduced.

Due to their controlled vocabulary and the clearly defined relations between terms, ontologies are useful to analyze large scale data or can be used to correlate data of different sources. For instance, the RNAi phenotypes of the ongoing iBeetle project and the mutant phenotypes stored in the Göttingen-Erlangen-Kansas-US Department for Agriculture (USDA) (GEKU) database [16] could be correlated. In addition, the correlation can be extended to the cross-species level [17]. *Tribolium* will be the first insect outside *Drosophila* with genome wide functional genetic data, which opens the possibility to do a comprehensive comparison of phenotypes between these two species.

In the biomedical field, ontologies are well established. The Gene Ontology (GO) [18] is the most important ontology in biomedical research, tagging genes with functional annotations and it is integral part of various kinds of automated data analyses and cross linking of data. On ontology portals like the OBO Foundry [12] or the NCBO Bioportal [19] anatomical ontologies form a major category. For the phylum of arthropods there are ontologies describing the morphology of a restricted subset of species like the Hymenopteran Anatomical Ontology HAO [14] or covers an extensive clade like the Arthropod Ontology AO. On the other hand, there are ontologies dealing with one species like the ontology FBbt [20] of the fruit fly *Drosophila melanogaster*. The latter is widely used for instance for the annotation of embryonic expression patterns at the Berkeley *Drosophila* Genome Project (BDGP) expression database and the annotation of neuroanatomy and protein expression of the brain [21]–[23].

In the scope of the iBeetle project, we have developed the *Tribolium* Ontology (TrOn) describing the morphology of *Tribolium castaneum* as first and only repository for the anatomical structures of the red flour beetle.

The role of TrOn in the iBeetle project is to support the screening procedure and most importantly to allow a semantic search function on the public web interface. The ontology makes it possible to annotate the most specific affected structure and at the same time allows searches for abstract terms comprising several concrete structures. Without an ontology, a user interested in searching for all walking leg phenotypes would have to combine searches for all its substructures with an OR-search. However, the TrOn ontology contains this information in form of the part_of relations, such that the user just needs to search for walking leg in order to find all phenotypes related to this structure and all it substructures.

Unfortunately, it was not reasonable to use one of the existing morphological ontologies and adapt it to *Tribolium* in a simple way. The fly ontology is very comprehensive and much of the adult beetle morphology can be aligned to *Drosophila* counterparts. However, there are also many differences, which are too significant to simply transfer the morphological features from one species to the other. For instance, the *Drosophila* leg is defined in the fly ontology as arising from imaginal discs during metamorphosis. While being correct for Dipterans, this is not true for *Tribolium* and most other insects, which do have legs already at the larval stage from which the adult legs develop. In contrast to the FBbt, the AO as well as the HAO are designed to comprise several species, but they lack most terms required to unambiguously identify specific morphological structures in *Tribolium*.

In this work we present TrOn, where most anatomical structures visible from the outside are annotated, defined and interconnected with part_of and is_a relations. With the help of the ontology based answers service (OBA service) [24] TrOn is already actively used in the public search interface of the iBeetle project. On the web pages of the iBeetle-Base the ontology can be browsed, searched and downloaded.

## Materials and Methods

The *Tribolium* anatomical ontology (TrOn) deals with the anatomical structures of the red flour beetle *Tribolium castaneum* at the developmental stages larva, pupa and adult. Where appropriate, the term hierarchy and definitions were taken from the *Drosophila* ontology (FBbt) or the Common Anatomy Reference Ontology (CARO) ontology [25]. The definitions of *Tribolium* specific structures are based on the text book from Sokoloff, respective publications [8], [11], [12] and the Handbook of Zoology [26]. The ontology is available in the OBO format [15] from the iBeetle-Base web page and the NCBO BioPortal.

The terms, relations and definitions where annotated with the ontology editor OBO-Edit in Version 2.3 [27]. For the OBA service, a plugin was developed to identify the concrete, mixed and generic terms and to provide search functions based on these sets. In the ontology, the three categories are represented as subsets which are retrieved from the OBA service.

**Figure 2:**
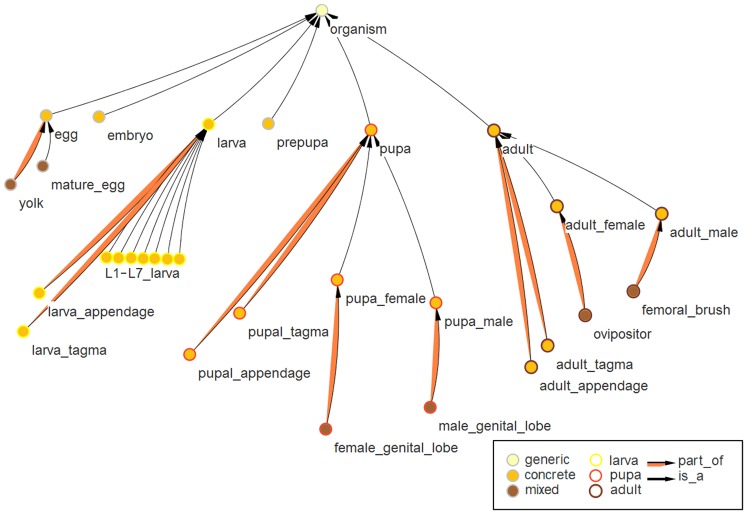
Representation of developmental stages in TrOn. This figure, exported from the ontology viewer OntoScope, displays the terms downstream of organism. These terms represent the red flour beetle at a specific developmental stage, which in nature are separated by molting (e.g. larva, pupa, adult). Transition stages, during which dramatic changes of morphology occur (i.e. prepupa and embryo) are included in TrOn to cover all developmental stages but were not linked with morphological structures. The is_a relations connect the terms with its parents (see thin black arrows). The anatomical structures are linked with part_of relations to the life stages (see orange arrows). The larval stages are divided into sub stages (L1–L7), which inherit the part_of relations from the parent term larva. Hence, the morphological structures are defined only once for the larval stage. The color of the center of the nodes represent the subset of the ontology the node belongs to, i.e. generic, concrete or mixed class. The border of the node indicates the developmental stage of the represented term.

**Figure 3:**
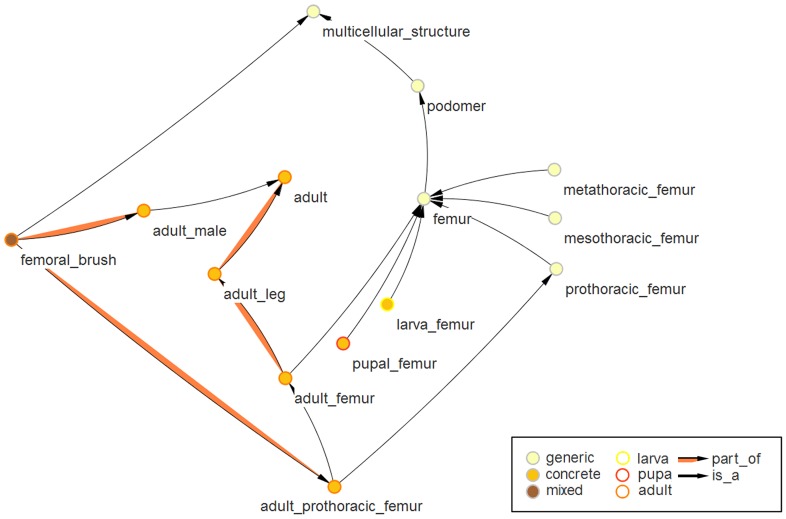
Generic and concrete subsets of terms. Selected terms and relations with respect to the femur are shown. The femur is the third leg segment (or podomer) of insects. It is present at all life stages, but looks quite different between e.g. larval and adult stages. Hence, femur is defined to be a generic term, i.e. an umbrella term, which defines morphological concepts, that are not linked to a certain stage or species. The concrete terms in contrast define dissectible structures of a certain life stage of *Tribolium*, which can be very different between life stages or insects species. The center’s colors of the nodes represent the subset of the ontology the node belongs to, i.e. generic, concrete or mixed class. The border of the node indicates the developmental stage of the represented term.

For the ontology viewer OntoScope [28] a plugin was developed to visualize TrOn which uses color codes to display the subset of a term and the developmental stage the term belongs to. OntoScope is used for the Figures 2 and 3 and is also available as Java webstart program from the iBeetle-Base web page.

## Results

### Defined Morphological Structures

The knowledge domain of the newly created *Tribolium* Ontology (TrOn) is the morphology of the red flour beetle *Tribolium castaneum* at the developmental stages larva, pupa and adult. The ontology comprises most morphological structures visible from the outside and some internal structures that are being screened during the iBeetle project. These structures are most of the somatic muscles, the stink glands and the ovary. Apart from stage specific structures, all entities are modeled for all appropriate life stages in the ontology. Substructures and connected structures are considered in all modeled life stages. Symmetrical structures are annotated once, not distinguishing between the left and right side. For symmetric and other multiple structures the singular form is used as name.

### Represented Life Stages

Besides of single structures and groups of them, TrOn contains ontology terms representing the beetle organism at each life stage. The anatomical structures specific for a developmental stage are linked with part_of relations to their stage, such that the ontology term representing a developmental stage is a collection of anatomical structures. The development of the beetle can be clearly divided into distinct stages separated by molts. These stages share common anatomical structures like legs or antenna. The anatomy of these stages, despite a common function, can be quite dissimilar at different stages. Consequently, we created for each structure in each developmental stage separate ontology terms. The terms are named in the same way for each stage (e.g. larva_leg, adult_leg). This holds true for concrete morphological structures like pupa_femur as well as for more abstract terms like larva_appendage.

The seven larval stages are quite similar to each other while pupal and adult morphology differs greatly. Therefore, the structures were not modeled separately for each larval stage but all structures are linked to the main term larva from where the relations are inherited by the subdivisions L1 to L7. These larval stages are specializations of the more abstract larva stage and therefore subterms of it. In addition, there are intermediate developmental stages, where dramatic changes in morphology occur (embryogenesis in the egg and metamorphosis during the pre-pupal stage). These stages are represented in the ontology but do not have morphological structures assigned to them because the respective dynamic processes are difficult to define using the static anatomical definitions of an anatomical ontology. The developmental stages are children of the ontology term organism, which by itself is a specialization of multicellular_structure (Figure 2).

### The Part_of Relation Type

In addition to the mandatory is_a links, the relation type part_of is used in TrOn. This relation type is not used in a strictly spatial sense, but also in a functional one, and is not defined as transitive in our ontology [29]. In most cases, the subterm is also a spatial subpart of the larger structure like the femur is a spatial part of the leg. On the other hand, the antenna is part_of the head but it is discussable if the antenna is inside the space of the head or just appended to the head and functionally related to it. A head without an antenna is still noticed as a head. The flagellum is part of the antenna but does not inherit the functional part_of relation of antenna to head, because the part_of relation type is not defined to be transitive in this case.

### Introducing Term Categorization Depending on their Relation to Life Stages

As a feature in TrOn, we introduce the categorization of all terms into concrete, generic and mixed categories (subsets), depending on their relation to a developmental stage. *Concrete terms* are linked to a specific developmental stage and represent anatomical structures that can be, at least theoretically, dissected from a beetle (e.g. the adult_femur). Upstream of the concrete ontology terms are the *generic terms*. These are independent of a developmental stage and represent the functional concept of a morphological structure (e.g. the femur), which is found at several stages or in several structures of one stage (e.g. sense_organ, which is found in many copies on the cuticle). Hence, generic terms usually comprise several, dissectible structures, i.e. they are umbrella terms. The third category subsumes terms of morphologically concrete structures, which are found only at one developmental stage, like the oocyte (female adult), the gin-trap (pupa) or the femoral_brush (male adult). These terms are categorized as *mixed terms* because they combine the characteristics of the generic and concrete category in a single term. Mixed terms are a subset of the concrete terms, because they are dissectible structures of a certain developmental stage. The naming of the mixed terms (i.e. concrete structures, which occur only at one stage) differs in that the stage is not part of the name.

The link of a term to a certain life stage can be direct or indirect. The part_of relation to a stage can be inherited through the is_a relation from the parents. Also the part_of relation type is used in a transitive way in the case of the assignment of a concrete term to a developmental stage. For instance, the adult femur is categorized as adult because of the two part_of relations between adult_femur and adult_leg and between adult_leg and adult. The categorization and the respective links and naming principles are shown for the term femur in Figure 3.The colors of the nodes represent the category they belong to. The generic term femur describes the third leg segment (or podomere) of any insect leg. It has children deduced by one of the following specializations. The first differentia is the leg to which the femur belongs, leading to the three children prothoracic_femur, mesothoracic_femur and metathoracic_femur. A distinction between left and right legs is not made because the corresponding structures are symmetrically equivalent. The second differentia added to the abstract term femur is the developmental stage. In TrOn this results in larva_femur, pupal_femur and adult_femur. The terms of the next level combine the two differentia of the first step. The concrete term larva_mesothoracic_femur has the two generic terms larva_femur as well as mesothoracic_femur as parent.

### Term Definitions

Most of the terms of TrOn were tagged with definitions, which help the user to understand the meaning of the respective term. External sources of information, which were used as base for the definitions, were referenced. We used the definitions of the Arthropod Ontology where possible, the textbook by Sokoloff and the Handbook of Zoology [26] for *Tribolium* specific structures and tried to match the definitions used in FlyBase anatomical ontology where sensible. An expert in insect anatomy was involved in order to finalize the definitions (S.Bradler). Table 1 summarizes the numbers of terms, the respective subsets and the number of relations of the current version of TrOn.

**Table 1:** Statistics of TrOn.

	Number
All terms	956
Cross references to other resources forall terms	111
Generic terms	306
Concrete terms	650
Mixed terms	54
Terms with a definition	951
Cross references to other resources forall definitions	2096
part_of relations	692
is_a relations	1373

### Application of TrOn for a Semantic Search for Morphological Defects at iBeetle-Base

In the ongoing genome wide RNAi screen iBeetle, morphological defects are annotated using a controlled vocabulary. As annotation guideline, the most specific structure affected was to be annotated. For example, when only the most proximal part of the walking leg was affected by a knock down, the corresponding term coxa was used. If the entire leg including the coxa was affected, the less specific term leg was used. As consequence of this annotation guideline, any simple search using the term “leg” would not identify the more restricted defects e.g. in the coxa or other substructures. Hence, a comprehensive search for all leg defects would have required a complex search combining all substructures with OR, which is not intuitive and error prone.

**Figure 4:**
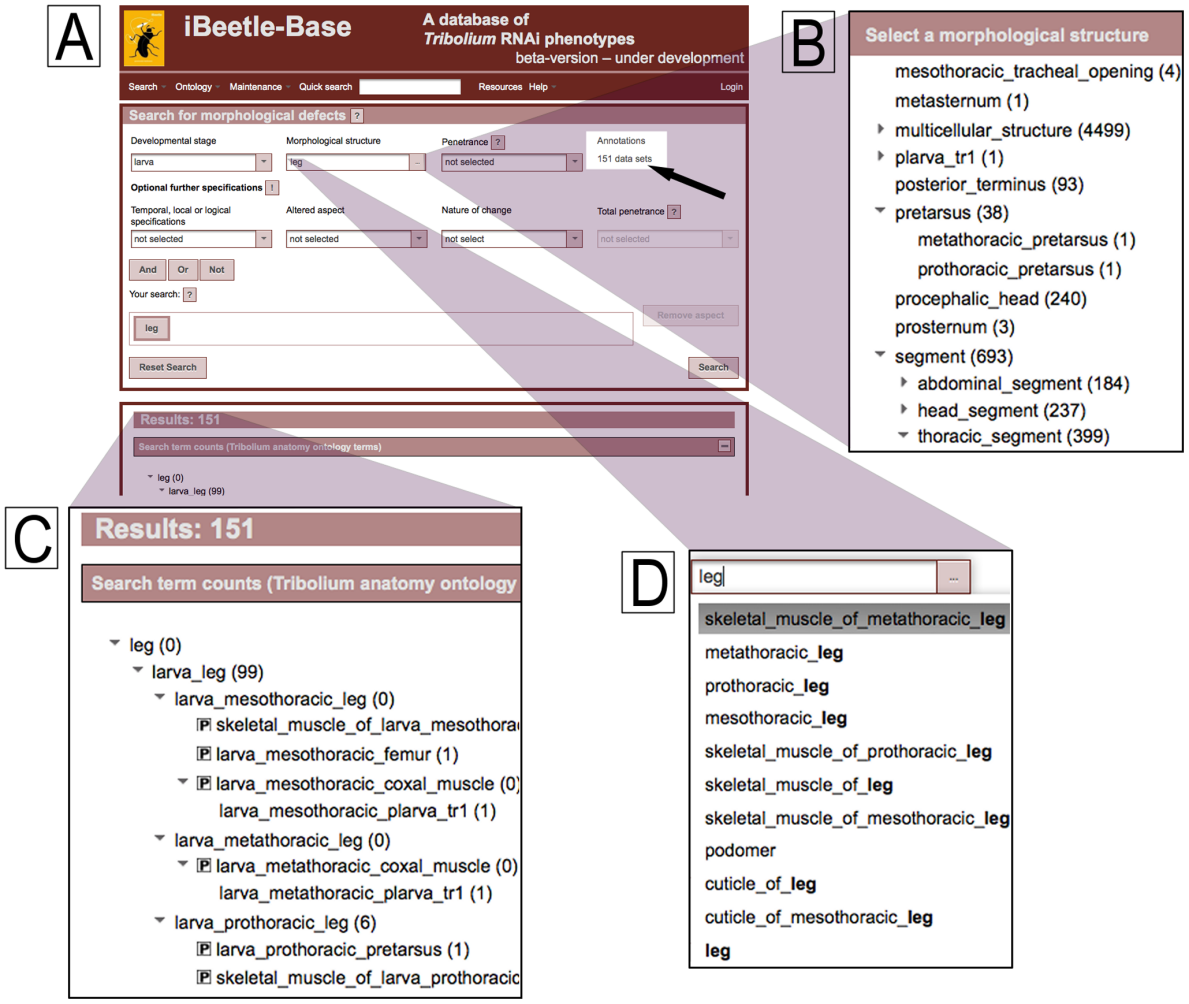
Application of TrOn at the iBeetle-Base. The figure shows a survey and detail views of the search interface of iBeetle Base that can be found at http://ibeetle-base.uni-goettingen.de. A) An overview of the search page is depicted. The black arrow points to the number of search results for the entered search terms. B) A suggestion tree is displayed upon click on the respective button, showing the generic terms of the ontology with annotated features of the iBeetle screen downstream. In brackets, the number of occurrences of a given term in the database is given. C) After a search, an extract of the ontology can be displayed, which represents the terms which contributed to the search result. In this case the number in brackets indicates the number of hits for this term in the search results. D) Upon typing a search term, a completion list is displayed. The displayed terms are generic terms which match the entered term or its synonyms.

In order to implement a comprehensive search, the terms of the controlled vocabulary are mapped, in conjunction with the developmental stage, to the concrete terms of the ontology. The relations documented in the ontology allowed finding all phenotypes affecting e.g. the leg and its substructures at all stages by just using the search term “leg”. An example is given in Figure 4 where the list of substructures and the number of respective annotations found in a search for “leg” is shown. After a term has been entered in the search field, the number of annotations with respect to this term and its children is given (see black arrow in Figure 4A). This helps the user to quickly assess and optimize the search with respect to specificity.

In detail, the ontology is traversed downstream of the search term in order to identify all of its successors. The search function uses the term hierarchy as well as the has_part relationship for the walk downstream. The has_part relations are not annotated in the ontology but are transiently generated by the OBA service as the inverse relationship to the annotated part_of relation type. The result of the search in the ontology is a list of concrete terms downstream of the search term. If a developmental stage was specified as filter, the search result consists only of the concrete terms, which are linked to this stage.

Another application of the generic terms of TrOn in the search interface of iBeetle-Base is to assist the user to find the appropriate search term. Upon typing a term into the search field, a completion list is displayed, based on the hits in the generic terms (see Figure 4D). As alternative means to identify a search term, an extract of the ontology tree can be displayed, which shows generic terms, the children of which (concrete terms) have been annotated in the screen (see Figure 4B). Together, the completion list and the suggestion tree support the user in finding the right name and level of abstraction for searching the anatomical structure.

After a search, the result list is accompanied by an extract of the ontology with all ontology terms which contributed to the result list. The number next to the term indicates the number of occurrences of the respective term in the results (see Figure 4C).

The OBA service is used to access the ontology in the web application. An OBA module for the iBeetle project implements the described functions and encapsulates the logic to process the ontology in the OBA service.

## Discussion

The *Tribolium* Ontology consists of nearly one thousand ontology terms describing most of the external morphology and a, so far, limited set of the internal structures of the red flour beetle. This focus reflects its initiation from the iBeetle project. However, the structures were defined more comprehensively than required for the iBeetle project and include substructures as well as developmental stages which are not annotated in the screen. While most of the external morphology is, hence, represented in TrOn, our systematic approach will allow expanding the ontology as the need arises. For instance, in contrast to the external morphology, internal structures like the central nervous system, musculature and digestive systems were not included or limited to subsets. The public availability of the ontology and the open OBO format will allow the community to adopt and use TrOn for future *Tribolium* projects. The team of iBeetle Base will endeavor to incorporate all comments and extensions of the community to keep a current and central knowledge resource about the red flour beetle.

Our effort to provide definitions for each term will help to use TrOn for additional purposes beyond a controlled vocabulary or a classification of morphological structures. For instance, definitions can be used to add information to applications and web sites which deal with morphological structures of *Tribolium* but do not require functions like the semantic search. TrOn as central repository of morphological terms and definitions may also help to agree on common definitions of morphological structures and will help to avoid divergent definitions of the same structure like the example of the paramere of the Hymenoptera shown by Yoder et al. [14].

Other important extensions to be included in future versions of TrOn are additional relation types. Corresponding structures of the different developmental stages can be connected with the relationship develops_to (e.g. the larva_leg develops_to the adult_leg passing through the pupal_leg). A develops_to relation type will help to clarify situations where one structure gives rise to two new ones and to unambiguously define development of the beetle for electronic systems. For instance, the single structure larva_tibiotarsus develops into the separate structures adult_tibia and adult_tarsus. Other possible relationships like attached_to, bordered_by would describe the spatial relations in a more sophisticated way. Functional aspects could be introduced with relations like innervated_by to denominate the target structures of nerves. Additional facets of the part_of relation could assign structures to physiological systems like the fat body or processes like communication and olfaction.

One of the most exciting possibilities opened up by ontologies describing the morphology of different insects is their use for automated cross species comparisons [17]. Together with the prospect of a genome wide analysis of gene function in *Tribolium*, this will allow for the first time to automatically compare the function of genes across insect taxa. In order to realize this potential, the terms of the *Tribolium* ontology need to be linked to the corresponding term of the ontologies in other insects. The basic architectures of the insect bodies have a common blueprint but during evolution, body parts have diversified their morphologies and functions. The wings serve as one example: Ancestrally, winged insects have two pairs of membranaceous wings used for flight. Both *Drosophila* and *Tribolium* have only one pair of membranaceous “wings”. In both cases the membranaceous wings are used to generate the required uplift and drive for flight. Also the morphology is comparable, they consist of a double layer of cuticle, and both have a complex and specific pattern of veins. However, these functionally and morphologically comparable structures are located on different segments. In the beetle, the membranaceous wings develop on the third thoracic segment, while in *Drosophila* they belong to the second thoracic segment. With the elytra (second thoracic segment of beetles) and the halteres (third thoracic segment on dipterans) both species have modified the other pair of wings both functionally and morphologically. Hence, crosslinking based on equivalent function of structures may lead to different connections than links based on homology of the structures (i.e. common evolutionary origin). Due to such functional evolution of structures, the cross link between the anatomical ontologies TrOn and FBbt need to be based on the evolutionary criterion of common descent (i.e. homology of the structures). See Bertone et al. for an effort to link the ontologies of different species [17].

In contrast to most anatomical ontologies of insects, in TrOn nearly all morphological structures have a generic term and a separate term for each developmental stage. This emphasizes the different morphology of insects at their different life stages. Upstream of the concrete terms, those that are linked to a development stage, the generic term represents the concept of the morphological structure independent of any stage. This conceptual separation allows unequivocally labeling the unique structure of a given insect with concrete terms, while the generic terms enable cross reference to other ontologies.

A major benefit of a cross linking between ontologies of different species is, among others, the possibility of a phenotypic search across several species. An example is the interconnection of fish phenotypes in the Phenoscape Knowledgebase [30]. However, to realize this aim, a mapping of the morphological ontologies is not sufficient. In addition, also the nature of morphological phenotypes needs to be mapped by a phenotypic ontology, like the Phenotype and Trait Ontology (PATO) [31]. Due to the availability of phenotypic data on a genome wide scale, a comparison between *Drosophila* and *Tribolium* represent a unique opportunity.

TrOn is already at its current stage a useful resource for the *Tribolium* research and a fundamental part of the iBeetle Base. Future priorities for the ontology are: (1) more relations types to describe functional and further connections (e.g. develops_to) (2) extend the scope of the covered internal morphological (3) cross-connection to other ontologies like FBbt from FlyBase and other insect ontologies.
